# Remodeling of the endothelial cell transcriptional program via paracrine and DNA-binding activities of MPO

**DOI:** 10.1016/j.isci.2024.108898

**Published:** 2024-01-12

**Authors:** Ruiyuan Zheng, Kyle Moynahan, Theodoros Georgomanolis, Egor Pavlenko, Simon Geissen, Athanasia Mizi, Simon Grimm, Harshal Nemade, Rizwan Rehimi, Jil Bastigkeit, Jan-Wilm Lackmann, Matti Adam, Alvaro Rada-Iglesias, Peter Nuernberg, Anna Klinke, Simon Poepsel, Stephan Baldus, Argyris Papantonis, Yulia Kargapolova

**Affiliations:** 1 Department III of Internal Medicine, Heart Center, Faculty of Medicine and University Hospital of Cologne, 50937 Cologne, Germany; 2 Cologne Center for Genomics (CCG), University of Cologne, 50931 Cologne, Germany; 3 Center for Molecular Medicine Cologne (CMMC), University of Cologne, 50931 Cologne, Germany; 4 Institute of Biomedicine and Biotechnology of Cantabria (IBBTEC), University of Cantabria, 39011 Santander, Spain; 5 Cluster of Excellence on Cellular Stress Responses in Age-Associated Diseases (CECAD), University of Cologne, 50931 Cologne, Germany; 6 Institute of Pathology, University Medical Center Göttingen, 37075 Göttingen, Germany

**Keywords:** Molecular mechanism of gene regulation, Immunology, Cell biology, Proteomics, Transcriptomics

## Abstract

Myeloperoxidase (MPO) is an enzyme that functions in host defense. MPO is released into the vascular lumen by neutrophils during inflammation and may adhere and subsequently penetrate endothelial cells (ECs) coating vascular walls. We show that MPO enters the nucleus of ECs and binds chromatin independently of its enzymatic activity. MPO drives chromatin decondensation at its binding sites and enhances condensation at neighboring regions. It binds loci relevant for endothelial-to-mesenchymal transition (EndMT) and affects the migratory potential of ECs. Finally, MPO interacts with the RNA-binding factor ILF3 thereby affecting its relative abundance between cytoplasm and nucleus. This interaction leads to change in stability of ILF3-bound transcripts. MPO-knockout mice exhibit reduced number of ECs at scar sites following myocardial infarction, indicating reduced neovascularization. In summary, we describe a non-enzymatic role for MPO in coordinating EndMT and controlling the fate of endothelial cells through direct chromatin binding and association with co-factors.

## Highlights

• MPO translocates into EC nuclei to bind chromatin
• MPO chromatin binding causes localized decondensation
• MPO triggers a partial EndMT program independent of enzymatic activity
• MPO-KO mice exhibit reduced number of ECs at infraction scar sites

## Graphical abstract

**Figure:**
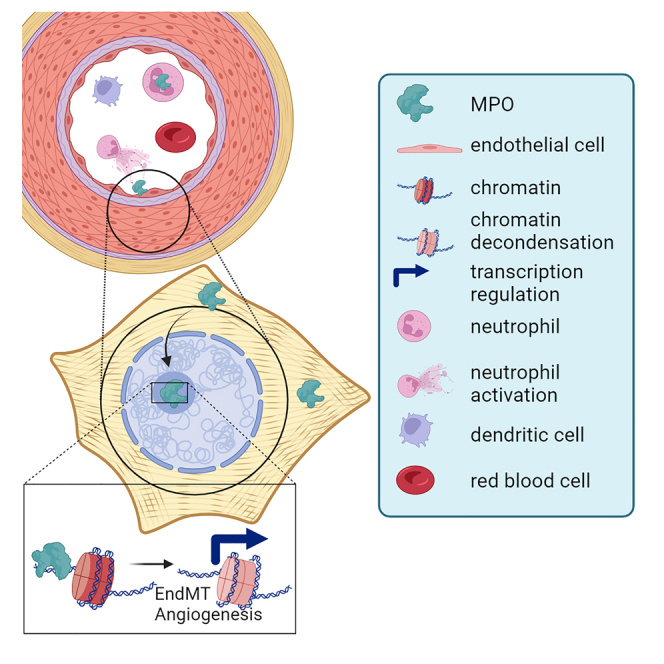

## Introduction

Myeloperoxidase (MPO), a lysosomal protein, functions in response to pathogens during bacterial and fungal infections and contributes to pathogen clearance within the phagosome. Catalytically active MPO uses hydrogen peroxide and halide or pseudohalide ions to catalyze production of hypochlorous, hypobromous, hypoiodous, or hypothiocyanous acids that are highly reactive and cause oxidation of proteins and small molecules.^1^^,^^2^ Multiple studies associated products of enzymatic activity of MPO with cardiovascular diseases, multiple sclerosis, cancer, and atherosclerosis.^3^

Apart from its microbicidal function, MPO has been shown to modulate endothelial cell (EC) surface integrity and EC function by the reduction of NO-bioavailability.^4^ It also acts as an autocrine regulator of leukocyte function by modulating leukocyte activation and adhesion.^5^ Moreover, it delays neutrophil apoptosis *in vitro,*^6^ suggesting that it functions as a survival signal for neutrophils and as such may prolong the duration of an inflammatory response.

Along with the well-studied enzymatic activity of the protein, MPO possesses non-enzymatic properties. First of them is an intrinsic affinity to DNA. In the process of formation of neutrophil extracellular traps (NETosis), MPO, which is in normal conditions primarily found in granules of resting cells, translocates to the nucleus where it causes the citrullination of histones and chromatin decondensation.^7^^,^^8^^,^^9^ Second example of non-enzymatic activity of MPO has been demonstrated by Manchanda et al.^10^ and was attributed to the cationic charge of the protein, causing a collapse of the endothelial glycocalyx.

Apart from its activity on the EC surface, MPO has a capacity to enter endothelial (EC) and epithelial cells.^4^^,^^11^ In less than 6 h, MPO can be detected in the cytoplasm and the nucleus of ECs, presumably remaining enzymatically active.^12^ However, ∼40% of the MPO is inactive at the site of inflammation.^13^ For instance, studies have shown the presence of 16–29 mg/mL of enzymatically inactive MPO (iMPO) in arthritic joints.^14^ Interestingly, both, inactive and active MPO can drive transcriptional changes in ECs.^15^ Among others, the expression and secretion of cytokines interleukin (IL)-6 and −8, MCP-1, and GM-CSF increases rapidly and in a dose-/time-dependent manner following MPO treatment.^15^ These findings suggest that MPO might affect gene expression via activity-independent mechanisms. Provided an affinity for chromatin, it may act as a transcriptional activator or repressor via direct regulation of chromatin organization. In spite of accumulating evidence supporting its non-enzymatic function on gene expression, the exact mechanism of how MPO acts, remains unknown. Here, we sought to mimic the circulating levels of MPO occurring *in vivo* in inflammatory conditions by adding MPO to ECs *in vitro* and thereby shed a light on the nuclear roles of MPO, likely constituting the first example of an enzyme contributing to cell-to-cell communication by entering the nucleus and directly binding chromatin to regulate gene expression.

## Results

### MPO accumulates in endothelial cell nuclei within hours

**Figure 1:**
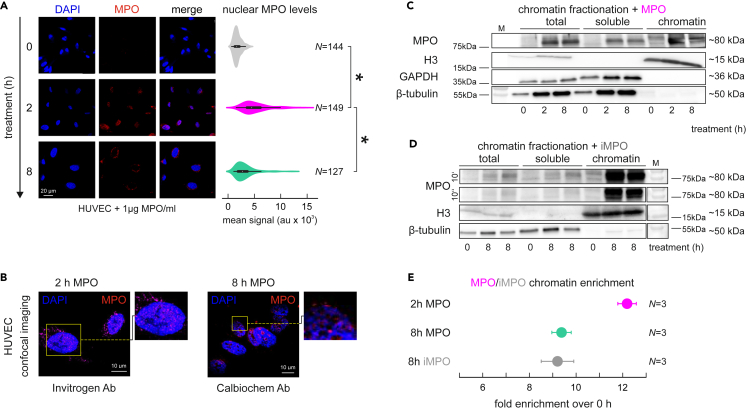
MPO translocates into ECs and binds chromatin already within 2 h after treatment (A) Representative images with immunofluorescence signal of MPO in ECs after 0, 2 and 8 h of MPO added to the media, quantification of mean nuclear MPO signal (right panel) calculated for 10–15 random images of three biological replicates in form of violin plots, *N* – number of individual nuclei; scale bar = 20μm. Statistical significance was determined by two-sample t-test. (B) Confocal image of MPO translocation in ECs upon 2- and 8-h MPO treatment using two different antibodies for IFs in at least 3 biological replicates; scale bar = 10μm. (C) Western blot determining total, soluble, and chromatin-bound MPO levels in ECs treated 2 or 8 h. (D) Western blot determining total, soluble and chromatin-bound levels of MPO in ECs treated 8 h with iMPO; images taken for 10 s or 10 min; 2 independent replicates are depicted. (E) Quantification of chromatin-bound MPO and iMPO enrichment compared to untreated cells and normalized to histone H3 levels; *N* = 3 biological replicates. Data are represented as mean ± SEM.

To assess MPO transmigration into EC nuclei, we treated human umbilical vein ECs with MPO for 2 and 8 h and performed immunofluorescence and fractionation western blot experiments. Accumulation of MPO in the nuclei of ECs 2 h after treatment was microscopically apparent (Figures 1A and 1B). This was reflected in the respective quantified mean nuclear signal intensities measured in >100 cells (Figure 1A). At 8 h post-treatment, nuclear MPO levels were ∼40% less than those at 2 h (Figures 1C and 1E). Notably, nuclear accumulation of MPO resulted in its presence on the chromatin fraction already at 2 h post-treatment (Figure 1C). Catalytically-inactive MPO was similarly enriched in the chromatin fraction of HUVECs (Figures 1D and 1E), indicating that this occurs independently of catalytic activity.

We sought to exclude potential effects on ECs caused by the MPO enzymatic activity through activation of autophagy by accumulation in lysosomes^16^ or of signaling pathways on the cell surface during its translocation into the nucleus.^17^ Western blot analysis of key autophagy factors revealed no difference in their levels (i.e., ATG5, ATG12, ATG7 or LC3A/B in Figures S1A and S1C). Furthermore, levels of latent monomeric TGFβ remained unaltered upon MPO treatment, and expectedly increased in hydrogen peroxide-treated ECs (Figures S1A and S1C). A mild decrease of phospho-SMAD, rescued by the addition of catalase, and no alteration in phospho-ERK levels were observed after MPO treatment (Figures S1B and S1C). This indicates that MPO-driven effects on transcription regulation of ECs likely happen independently of signaling pathways, affected by MPO treatment.

In light of the fact that the precise nuclear localizing domain of the MPO protein has not yet been determined, our study aimed to identify this domain by fusing different segments of MPO with an mVenus protein.^18^ We divided the MPO protein into three segments of approximately equal size: the N-terminal segment consisting of 252 amino acids, the middle segment consisting of 256 amino acids, and the C-terminal segment consisting of 237 amino acids. These segments were then fused with the mVenus protein. To generate stable and doxycycline-inducible cell lines, we transfected HEK293T cells with these fusion constructs.

Upon induction with doxycycline in the stable cell lines expressing the N-terminal and middle segments of MPO, we observed only marginal levels of mVenus signal in the nuclei, while a strong signal accumulated in the cytoplasm (Figures S1D and S1F). The preferential cytoplasmic localization was also observed for the full length MPO protein in HEK293T overexpressing cells. It may be attributed to the absence of modifications and adequate maturation processes, which are exclusive to neutrophils. However, fusion of mVenus with the C-terminal segment of MPO resulted in an increased fluorescence signal in the nucleus (Figures S1D and S1F). Interestingly, when we combined the C-terminal segment of MPO with either the N-terminal or middle segment, but not both together, we observed an enhanced nuclear mVenus signal (Figure S1G).

This observation suggests the potential presence of a nuclear localizing domain in the C-terminal region of MPO. In order to identify this domain more precisely, the C-terminal portion of MPO was fragmented into four peptides, each consisting of 68, 67, 44, and 58 amino acids in length. These peptides were then fused with mVenus, as shown in Figure S1E. The results revealed that the two most C-terminal regions of MPO exhibited a predominantly nuclear accumulation of fluorescence (Figures S1E and S1H). To further investigate the specific region within the C-terminal part of MPO that may function as a nuclear localization signal (NLS), we employed the NLStradamus^19^ tool. Through this analysis, we identified the KRKGR motif (amino acids 652–656) within the C-terminal part 3 of MPO as the most promising candidate for an NLS. Based on these findings, we propose that the MPO NLS is primarily located within the last 102 amino acids of the C-terminal region of the MPO protein, which may include, but is not limited to, the KRKGR motif.

### MPO directly binds chromatin at nucleosome-free regions

**Figure 2:**
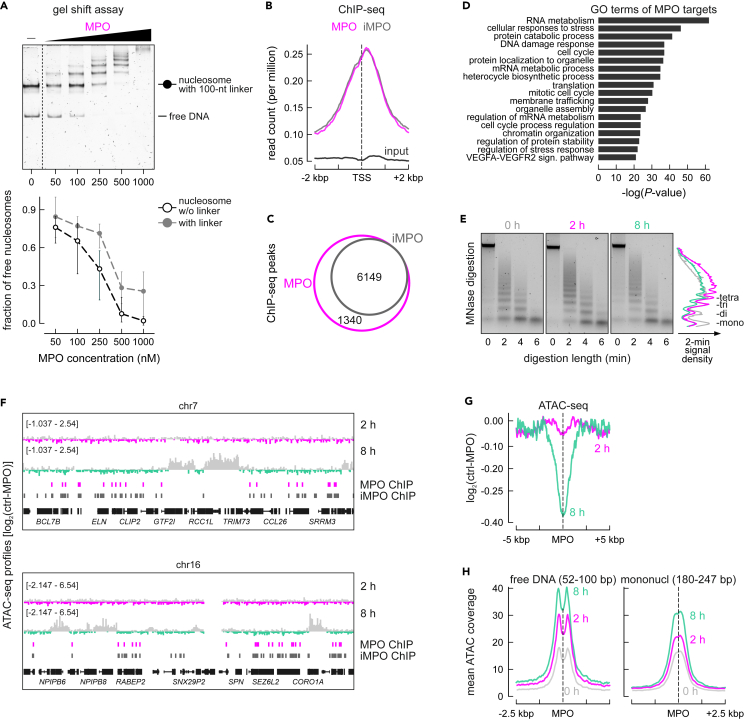
Myeloperoxidase binds to chromatin *in vitro* and *in cyto*, causing changes in chromatin condensation (A) EMSAs performed with increasing titers of MPO (50-1000nM) and nucleosomes with 100bp, long liker DNA or with 0bp, ‘no linker’ DNA. Quantification of the assay is shown in the lower panel, 3 replicas were analyzed. Data are represented as mean ± SEM. (B) Line plot showing mean distribution of MPO (pink) and iMPO (gray) ChIP-seq and input signal (black). (C) Venn diagrams showing overlap between conserved ChIP-seq peaks for MPO (pink) and iMPO (gray). (D) Significantly enriched GO terms associated with sets of 3000 genes, bound by MPO (within 670 bp of TSSs). (E) Electrophoresis profiles of DNA fragments released by MNase from ECs treated with MPO for 0, 2 and 8 h. Signal density after 2 min of MNase treatment was quantified and plotted as a line-plot (right panel) – mono-, di-, tri, and tetra-nucleosomes are labeled; experiment performed in triplicates. (F) Representative genome browser views of ATAC-seq coverage-based log2 ratios of control vs. 2-h or control vs. 8-h MPO-treated chromatin merged with positions of MPO and iMPO ChIP-seq peaks. (G) ATAC accessibility in the 10 kbp around MPO-bound positions calculated as log2 ratios of control vs. 2-h MPO (pink) or control vs. 8-h MPO treatment (green). (H) ATAC accessibility in the 5 kbp around MPO-bound positions for a subset of reads between 52 and 100 bp, corresponding to free DNA (left) or between 180 and 247 bp, corresponding to mononucleosomes (right).

To estimate the propensity of MPO to bind nucleosomes *in vitro* electromobility shift assays (EMSA) were performed, in which MPO was titrated between 50 and 1000 nM to bind nucleosomes with ‘no’ (0bp) and ‘long’ (∼100bp) DNA linkers (Figures 2A and S2A). MPO displayed binding to long linker nucleosomes at low titers, and ‘no linker’ nucleosomes at medium to high titers (Figure 2A, lower panel). Binding was observed to be independent of enzymatic activity by inactivated MPO (Figure S2B). The decline in efficient binding can be attributed to the process of MPO inactivation, resulting in the loss of the heme group (Figure S2B).

The genome-wide capacity of chromatin binding by enzymatically-active and inactive MPO (iMPO) was assessed by chromatin immunoprecipitation and sequencing (ChIP-Seq; Figure 2B). To account for the transient nature of MPO binding to chromatin and the heterogeneity in MPO distribution (with some cells acquiring nuclear MPO at later time point) this experiment was performed at 8 h post-treatment using an antibody successfully applied in immunostainings and western blots. MPO and iMPO binding was mainly detected at transcription start sites (TSSs) (Figures 2B, S2C, and S2E). MPO/iMPO ChIP-Seq was performed in duplicates, with conserved peaks converging for active and inactive protein (Figure 2C; Table S1). For example, MPO/iMPO peaks co-localized with regions of open chromatin at the *KIT* locus (Figure S2D) and were overall enriched for POLR2A and H3K4me3 signal based on the analysis of ENCODE ChIP-Seq data (Figure S2F). This suggests a preference for TSSs of actively transcribed genes, but not for enhancer regions (Figures S2E and S2G). Gene ontology analysis performed via Metascape^20^ uncovered gene clusters related to cell cycle, chromatin organization, and VEGFA-VEGFR2 signaling (Figure 2D), all previously implicated in the process of angiogenesis.^21^

The consequences of MPO binding to chromatin were assessed via micrococcal nuclease digestion followed by electrophoretic analysis at different time points after treatment. These experiments indicated increased, time- and concentration-dependent chromatin condensation caused by MPO treatment (Figures 2E and S2H). To map sites of MPO-driven chromatin condensation, ATAC-seq was performed. Little change in chromatin accessibility (with a bias for increased condensation) was measured 2 h post-treatment and globally distributed, as exemplified for chromosomes 7 and 16 (Figure 2F). Longer MPO treatment caused profound condensation at specific regions and moderate de-condensation next to MPO binding sites (Figures 2F and 2G). The specific areas of moderately increased chromatin condensation were computationally identified as areas of 2-fold condensation in a 2-kbp or larger windows (Table S2). According to the nucleosome positioning analysis, MPO-bound regions are mostly nucleosome-free with binding close to the entry/exit site of the adjacent nucleosome. However, MPO binding did not cause any shift in nucleosome positioning (Figure 2H).

### MPO induces endothelial-to-mesenchymal transition to endothelial cells

**Figure 3:**
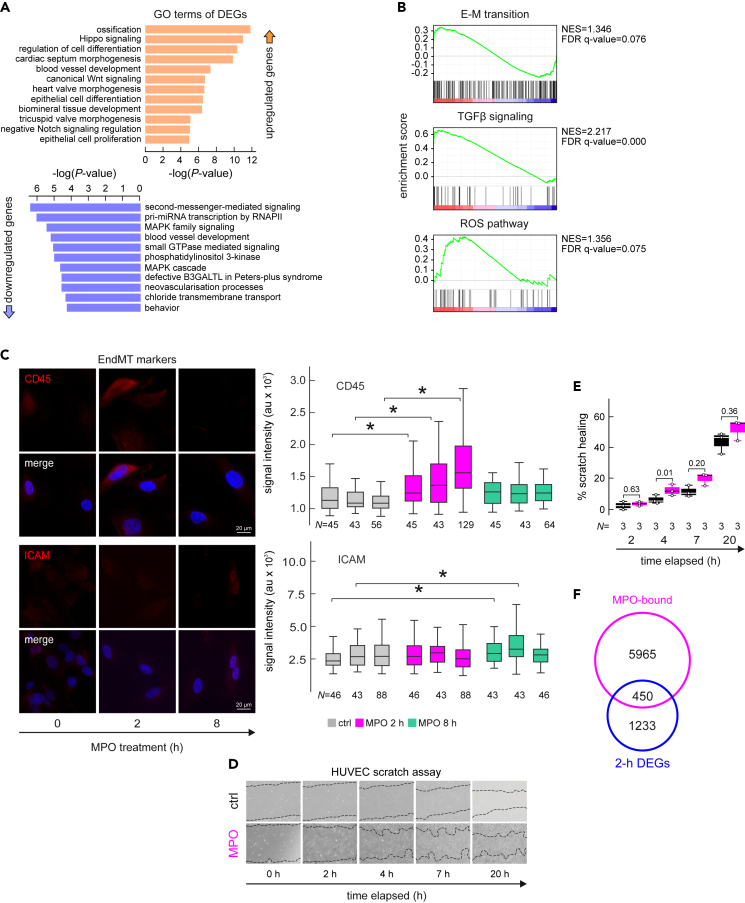
MPO triggers endothelial-to-mesenchymal transition in ECs (A) GO terms associated with up- (orange) or down-regulated (blue) genes upon 2-h MPO treatment. (B) Gene set enrichment analysis (GSEA) of ranked gene expression data, generated by RNA-seq of HUVECS under 2 h MPO treatment, FDR q-values <0.1. (C) Representative immunofluorescence images of CD45 and ICAM in HUVECs upon 0, 2 and 8 h of MPO treatment, scale bar = 10μm. Mean signal of total ICAM and CD45 plotted as violin plots – results of three biological replicates are summarized, N – number of individual cells, ∗ - t-test <0.05. Data are represented as mean ± SEM. Statistical significance was determined by two-sample t-test. (D and E) Wound healing assay performed after MPO (bottom row) or mock treatment (top row) over the period of 20 h, images taken at 0, 2, 4, 7 and 20 h, % of scratch healing calculated relatively to 0 time point, mean data of three biological replicates are plotted. Data are represented as mean ± SEM. Statistical significance was determined by Welch’s t-test. (F) Venn diagrams showing overlap between MPO-bound genes and differentially expressed genes (all signif. regulated, padj. < 0.05) at 2 h post treatment.

The impact of MPO treatment on gene expression was measured by RNA-seq at 2 or 8 h post-treatment compared to untreated ECs. Differential expression of 456 and 65 genes (fold change >2; Padj <0.05) was observed after 2 and 8 h of MPO treatment, respectively (Figure 3A), suggesting maximum degree of gene regulation coincides with maximum of chromatin-bound MPO (Table S3). GO term enrichment revealed ossification, blood vessel development and negative regulation of Notch signaling for the upregulated genes, and MAPK family signaling as well as chloride transmembrane transport for downregulated ones at 2 h after treatment (Figure 3A). Gene Set Enrichment Analysis (GSEA) revealed β-catenin signaling, adipogenesis, DNA repair and other pathways were shared by genes regulated at both 2- and 8-h post-treatment (Figures 3B and S3C). Treatment of ECs with enzymatically inactive mutant variant of MPO, Q91T^22^ allowed identification of only 42 differentially-expressed genes (fold change >2; Padj <0.05) of which 31 were also significantly regulated by enzymatically-active MPO treatment (Table S3). The reduced number of regulated genes observed in cells treated with inactive MPO may be attributed to the change in its tertiary structure, as a result of a dissociation of the heme group from the protein during inactivation or in the event of a mutation. Interestingly, expression of TGFβ1 gene was slightly upregulated (1.6-fold) upon 2 h of MPO treatment. This effect however was of a transient nature and at 8 h post-treatment no activation of TGFβ1 expression has been detected. Genes related to the Senescence Associated Secretory Phenotype (SASP) were regulated by both the active and the mutant form of MPO (Figure S3B).

When up- and downregulated genes were co-considered, GO and GSEA analyses suggested that ECs undergo endothelial-to-mesenchymal transition (EndMT) (Figure 3B). Selected markers of endothelial and mesenchymal cells were assessed by RT-qPCR (Figure S3A) and immunofluorescence (Figures 3C and S3D), and confirmed EndMT induction independent of enzymatic activity of MPO. Protein levels of CD45, an indispensable driver of the EndMT following myocardial infarction,^23^^,^^24^ increased 2 h post-treatment in ECs in three biological replicates (Figure 3C). CD34, an endothelial progenitor marker, was significantly downregulated 2 h post-treatment (Figure S3D), whereas CD31 levels (platelet/EC adhesion molecule-1 - PECAM-1) increased at 8 h post-treatment (Figure S3D). This shows that MPO triggers partial and transient EndMT to ECs.

A hallmark of EndMT is increased migratory potential. The wound healing/scratch assay, performed with and without MPO in the media, showed MPO treatment enhanced migratory potential of ECs. The observed changes could be interpreted as increased cell migration triggered by EndMT or other pro-migration factors like increased angiogenesis (Figures 3D and 3E).

In contrast to other factors implicated in EndMT, such as tumor necrosis factor alpha (TNFα), a proinflammatory cytokine that transmits signals through nuclear factor kappa B (NF-κB),^25^ the transcriptional signatures driven by MPO were distinct and did not exhibit significant overlap (Figure S3E). Only 32 genes were regulated by both treatments (two-fold change, Pval <0.05), and among them, 9 genes were reciprocally regulated (Table S3). Furthermore, we compared the transcription start sites (TSSs) bound by MPO (within <700 bp) and identified 450 genes that exhibited transcriptional regulation of 1.5-fold or more. Similarly, we intersected the genomic intervals undergoing chromatin condensation with the gene set regulated at 8 h post-treatment, and we annotated 136 sites located within or adjacent to differentially regulated genes (Padj <0.05). These findings collectively suggest that MPO can directly influence gene expression by inducing changes in chromatin structure, particularly in the vicinity of gene promoters.

### MPO interacts with ILF3 and affects its subcellular localization

**Figure 4:**
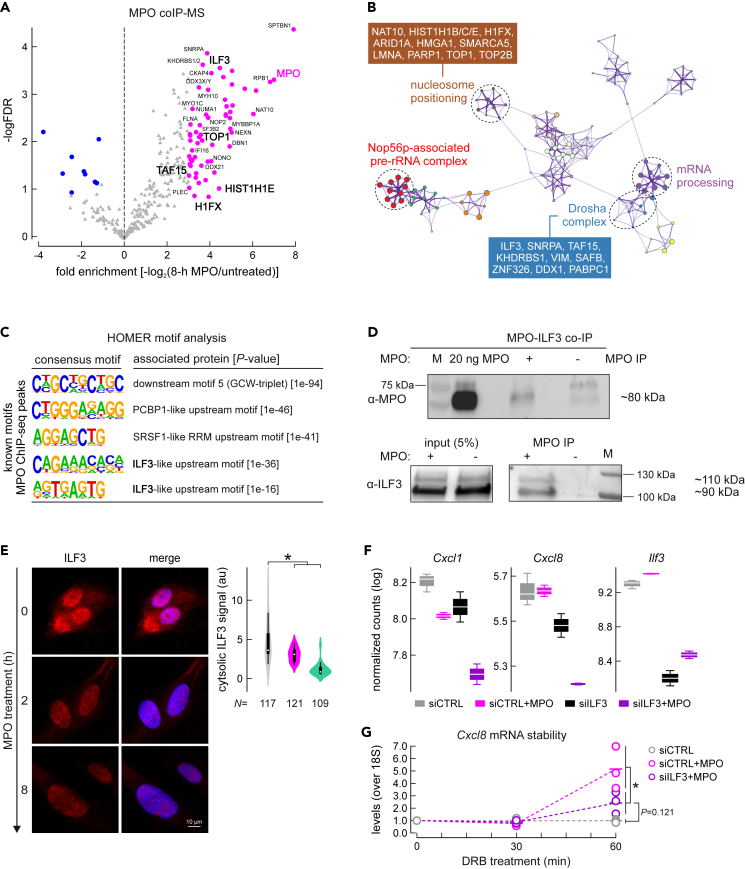
MPO binds to transcription factor ILF3 and regulates specific gene stability and expression (A) Volcano plot of MPO interacting proteins, significantly enriched factors are depicted in pink. (B) Metascape-based network representation of GO terms associated with proteins co-purifying with MPO, exemplified are proteins forming GO terms “Large Drosha complex” and “nucleosome positioning”. (C) Five most relevant motifs found with help of HOMER and within a 200 bp region around the center of MPO ChIP-Seq peaks, resembling motifs recognized by PCBP1, SRSF1 and ILF3 proteins. (D) Western blot visualization of ILF3 protein co-immunoprecipitated with MPO. (E) Immunofluorescence image depicting decrease of ILF3 signal in the cytoplasm of MPO-treated ECs, with quantification (right panel), scale bar = 10μm. Data are represented as mean ± SEM. Statistical significance was determined by two-sample t-test. (F) Boxplots, showing representative regularized log transformed counts of 3′-end sequencing experiment of *Cxcl1*, *Cxcl8* and *Vegfa* upon treatment with mock control siRNA, siRNA against ILF3, or a combination of siRNA treatments with MPO. Data are represented as mean ± SEM. (G) Stability assay performed with help of RT-qPCR and after inhibition of transcription with 5,6-Dichlorobenzimidazole 1-beta-D-ribofuranoside (DRB) for 1 h time, detecting levels of Cxcl8 transcript, and normalized by 18S. Statistical significance was determined by Welch’s t-test.

To determine interaction partners of MPO in EC nuclei, an immunoprecipitation of MPO from nuclei treated for 0 and 8 h was performed (Figure 4A). The MPO interactome was cataloged by mass spectrometry and visualized using Metascape. MPO interacts with proteins known to regulate mRNA processing and stability, nucleosome positioning, and pre-rRNA complexes (Figure 4B).

One of the RNA-binding factors identified amongst interacting partners of MPO (and confirmed by co-IP western blot) was ILF3 (Figures 4A and 4D). An analysis of motifs at MPO-binding sites revealed ILF3- and PCBP1-like motifs among the most plausible (Figure 4C), and both these proteins were identified as MPO-interacting partners (Table S4).

ILF3 is a well-characterized RNA-binding protein, with its smaller isoform (NF90) recently shown to also act as a transcription factor.^26^^,^^27^ ILF3 localization via immunofluorescence showed loss of cytoplasmic ILF3 signal at 2 and 8 h post-treatment (Figure 4E). Additionally, gain of NF90 in the chromatin fraction of ECs at 2 and 8 h post-treatment was detected (Figure S4A). Interestingly, a slight increase of NF90 in the chromatin fractions of ECs was also observed after treatment with the enzymatically inactive MPO variant Q91T (Figure S4A), suggesting that changes in ILF3 abundance were not linked to the enzymatic activity of the protein.

To determine the transcriptional network controlled by ILF3, we performed ILF3 knockdowns (with an efficacy of >70%; Figure S4B) followed by 3′-end sequencing of polyadenylated RNAs. ILF3 depletion caused significant changes to the levels of 110 genes (Padj <0.1; Table S5; Figure S4C). Interestingly, when MPO treatment was performed on top of ILF3 depletion, only 47 genes were differentially expressed (Table S5). Then, MPO treatment led to the differential expression of 37 genes when performed upon control siRNA transfection, whereas only 13 genes were significantly regulated by MPO after ILF3 knockdown, suggesting that partial regulation of genes via MPO is dependent on sufficient ILF3 levels.

Finally, we speculated that transcripts known for being stabilized by ILF3,^28^ e.g., *CXCL8* (Figure 4F) and *PPDPF* (Figure S4D), would be downregulated when ILF3 is depleted, but MPO treatment led to their stabilization. Thus, we measured transcript abundance after inhibition of ongoing transcription by DRB (5,6-Dichloro-1-β-D-ribofuranosylbenzimidazole). Monitoring the effects of DRB inhibition on *CXCL8* and *PPDPF* abundance for up to 60 min revealed that MPO treatment increased stability of both, whereas the combination of MPO treatment with ILF3 depletion significantly diminished this effect (Figure 4G and S4E). This finding supports that MPO might partake in stabilization of mRNA transcripts in ILF3-dependent fashion.

### *In vivo* role of MPO in neovascularization in a model of myocardial infarction

**Figure 5:**
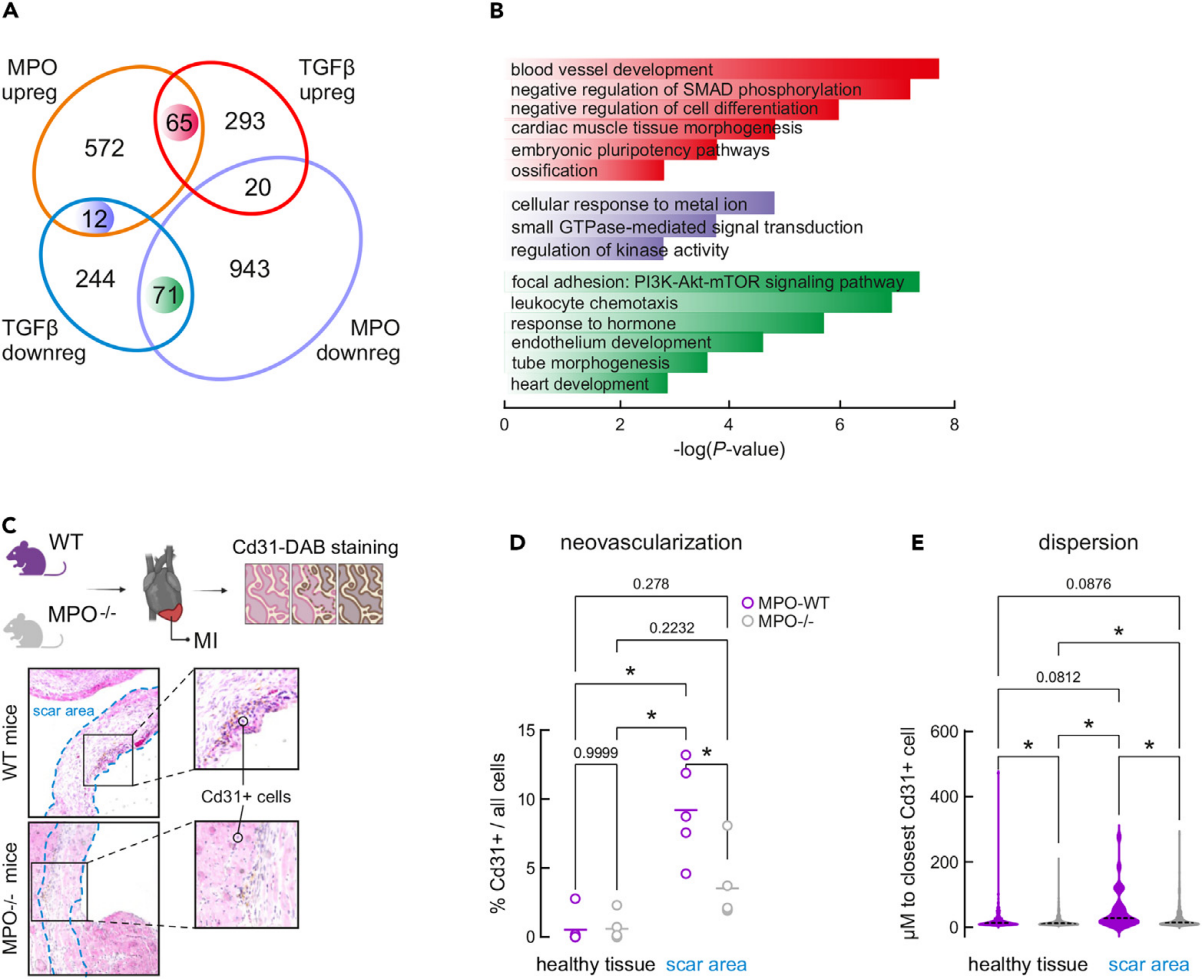
MPO and TGFβ regulate similar sets of genes involved in blood vessel development (A) Venn diagram depicting the overlap between gene sets, up- and down-regulated upon MPO (current study) and TGFβ treatment.^31^ (B) Barplot depicting top results of Metascape analysis of gene sets reciprocally or commonly up- or down-regulated by TGFβ^31^ and MPO (this study). (C) DAB and H&E staining applied to identify CD31^+^ cells. (D) Barplot showing the percent of CD31^+^ (a measure of capillary endothelial cells) as approximation for neovascularization in healthy and scar tissue of MPO^−/−^ and MPO^+/+^ animals after 8 weeks post myocardial infarction (two-way ANOVA test), ∗: p < 0.001. Each dot represents one animal, >50 images analyzed per animal. (E) Bar plot showing distance to the closest CD31^+^ in healthy and scar regions of MPO^+/+^ and MPO^−/−^ mice after MI, ∗: p < 0.001. Statistical significance was determined by two-way ANOVA test.

EC migration is essential for angiogenesis, a formation of new vessels from pre-existing ones. This process is regulated by a tight balance between pro- and antiangiogenic agents and involves a cascade of events, of which migration of capillary ECs is an essential component. A recent publication by Tombor et al. revealed that after myocardial infarction (MI) ECs acquire a transient state of mesenchymal transition in the first week after MI.^29^ Interestingly, inhibition of mesenchymal activation by the TGFβ inhibitor Galunisertib (LY2157299) reduced the incidence of clonal expansion, suggesting that mesenchymal activation contributes to endothelial expansion.^29^ Curiously, as a result of Galunisertib treatment, a reduced serum levels of MPO, IL-1β and IL-6 were observed in the acute pancreatitis model, implying those factors could also contribute to mesenchymal activation.^30^ We overlapped TGFβ regulated genes^31^ with those changes by MPO treatment, and observed activation of expression of 65 common genes and repression of 71 common genes by MPO and TGFβ in ECs, whereas 12 genes were reciprocally regulated (Figure 5A). Intriguingly, the cohort of common upregulated genes were enriched in category ‘blood vessel development’ (Figure 5B). TGFβ treatment of ECs significantly suppresses *Cxcl1* (log2FC −1.3; padj 0.0004) and *Cxcl8* (log2FC −2.0; padj 6.31e-08) and activates expression of *FN1* (log2FC 1.8, padj 3.34e-14) and *Edn1* (log2FC 1.18 padj 1,42e-07), level of *Ilf3* however only slightly decreases upon TGF-β treatment (S5A). Interestingly, genes bound by MPO directly and positively regulated by MPO and TGF-β are enriched in ‘Integrin 3 pathway’, ‘signaling by TGF-β family members’ and ‘EMT’, suggesting MPO can enhance activation of TGFβ signaling by direct chromatin-binding and gene activation (Figure S5B). Furthermore, MPO treatment causes 1.6-fold increase in TGFβ mRNA level at 2h post treatment, this regulation has a temporary nature and is diminished at 8 h post MPO treatment (Table S3).

To ask whether MPO similarly to TGFβ impacts neovascularization, we performed *in vivo* experiment, in which number of CD31^+^ cells, marking capillary ECs, were compared in MPO^−/−^ and MPO^+/+^ mice after 8 weeks post myocardial infarction (Figure 5C). The number of CD31^+^ cells was significantly higher in MPO^+/+^ animals at the scar area, whereas no difference observed for healthy tissue (Figure 5D). Furthermore, distances to the closest CD31^+^ cell was significantly larger in both healthy tissue and scar area of MPO^−/−^ animals (Figure 5E). This observation confirms that MPO might play additional roles in activating EndMT and enhancing cell-to-cell communication coordinated by factors such as TGFβ.

## Discussion

In the frame of this work, we have shown that MPO is being endocytosed and transmigrates through the HUVEC cells within a period of 24 h. MPO is a 150-kDa heterotetramer, consisting of two 15-kDa light chains and two ∼55kDa variably glycosylated heavy chains bound to a heme group. There can be several ways how MPO moves into the nucleus. It can move into the nucleus as a heterotetramer, especially since we already showed that being catalytically active is disconnected from its nuclear effects. The prediction of NLS-like region of MPO with help of NLStradamus, led to identification of two regions - the N-terminal locus comprised of amino acids 75–92 (KAYKERRESIKQRLRGS) and a C-terminal comprised of amino acids 641–673 (DIWMGGVSEPLKRKGRVPLLACIIGTQFRKLR). Experimentally we mapped the nuclear localizing domain of MPO to the C-terminal part of the protein, within the last 102 amino acids.

We posit that MPO protein translocation within ECs has a transient nature and contributes to the transient transcriptional changes. We observed that by the time of 2 h post-treatment, MPO is majorly found bound to the chromatin of ECs, and this binding is independent of the enzymatic activity of the protein. Similar capacity of MPO to bind chromatin has been observed during neutrophil activation.^9^ *In vitro* and *in cyto* experiments performed in frame of this study suggest MPO preferably binds to the nucleosome-free regions of the chromatin, and at transcription start sites of actively transcribed genes. Locally, at the sites of MPO binding, the chromatin becomes less condensed. This local de-condensation may change chromatin accessibility for specific transcription factors, thereby affecting transcription of more than four hundred genes. Interestingly, we have identified increased phosphorylation of histone H1.5 at the position 18 (pS18-H1.5) directly bound by MPO (Table S4). The regulation of phosphorylation of Histone 1 variants was previously associated with chromatin de-condensation and differentiation of pluripotent cells.^32^ On the other hand, regions flanking the MPO binding sites gain condensation, transcription at those sites gets mainly repressed (Table S2). Another mode of MPO-driven chromatin accessibility change can be accomplished via direct interaction with the chromatin remodellers, the SWI/SNF-ATPase subunit ARID1A and an ISWI-ATPase subunit SMARCA5 (Table S4).

During MPO treatment a wave of transcription regulation occurs, being more pronounced at 2 h, which coincides with the maximum of measured MPO bound to the chromatin. The transcriptional program triggered by MPO leads to partial endothelial-to-mesenchymal transition of ECs and is seemingly independent of enzymatic activity of the protein. EndMT is a type of cellular transdifferentiation, demonstrated by ECs with remarkable phenotypic plasticity. EndMT partakes in embryonic development and pathogenesis. During the disease of pulmonary arterial hypertension, EndMT is causative for structural and functional pulmonary vessel alterations, during atherosclerosis it leads to an increased vascular wall fibrosis, resulting in vessel narrowing and during fibrodysplasia ossificans progressiva – leads to soft tissue calcification.^33^ In our setup, the ECs undergo only partial EndMT. It is not driven by altered TGFβ levels, and is independent of NF-κΒ signaling.

Although TGFβ protein levels are not altered by MPO, the genes regulated by MPO and TGFβ partially overlap. In particularly, gene set positively regulated by both factors is enriched in ‘blood vessel development’. The loss of MPO *in vivo*, leads to reduced neovascularization after myocardial infarction, it partakes in mesenchymal activation of ECs *in vivo*, similar to TGFβ. The absence of differential expression of TGFβ implies that the process of EndMT is initiated by MPO. This is evidenced by the activity of MPO as a signaling molecule, as it enters the nucleus and binds to chromatin to modulate gene expression. While numerous factors that trigger EndMT have been documented, we suggest that MPO should be included in this comprehensive list.

MPO directly interacts with ILF3 protein and causes its depletion in the cytoplasm. MPO increases stability of ILF3-bound transcripts and does so in ILF3-dependent manner. ILF3 is a multi-faceted protein acting as an RNA-binding protein, implicated in a selective regulation of gene expression, mRNA stabilization of *Bcl2*, *IL2*, *MyoD*, *Cxcl1*, *Il8*, *Vegf*,^28^ translation inhibition of SASP factors,^34^ modulation of viral replication and noncoding RNA biogenesis.^35^ Both isoforms of ILF3 (NF110 and NF90) were found in the nucleus and cytosol and are shuttling between these two compartments, driven by post-translational modifications.^35^ Moreover, Reichman with colleagues^26^ discovered that ILF3 proteins were associated with chromatin, whereas Wu with co-authors performed chromatin immunoprecipitation of NF90/ILF3 from K562 erythroleukemia cells,^27^ to identify the positions of binding sites co-localized with chromatin marks associated with active promoters and strong enhancers. High expression of ILF3 was associated with angiogenesis in cultured human coronary artery endothelial cells (hCAECs), whereas knockdown in hCAECs reduced abundance of several angiogenic factors, and reduced proliferation rate of cells.^28^ We showed that observed effects of MPO on transcript abundance was dependent on high levels of ILF3, and diminished with ILF3 depletion. We speculate those effects were maintained via ILF3-regulated stability of given transcripts, when combined with MPO, it enhanced post-transcriptional regulation.

We observed that MPO drives regulation of SASP transcripts (Figure S3B). This type of regulation might be important during tissue regeneration. The potential role of SASP and senescent cells during wound healing was proposed by Lopes-Paciencia et al.,^36^ stating that transient exposure to SASP leads to increased regenerative capacity *in vivo*. Therefore, we speculate that the short exposure of ECs to MPO might be beneficial during the process of tissue regeneration.

The mouse model was employed to augment the physiological significance of our observations regarding MPO-induced alterations in gene expression in ECs. Our findings suggest that MPO may play a role in the neovascularization following myocardial infarction.

In conclusion, three modes of enzymatically independent activity of MPO on EC gene regulation were revealed by current work. These include direct effect on transcription, indirect regulation of transcription via events of chromatin condensation/decondensation and lastly, ILF3-dependent regulation of transcripts’ stability.

### Limitations of the study

It is important to note that our study’s limitation lies in the fact that we conducted RNA-sequencing and ChIP-sequencing to identify the regulon and the MPO binding sites at only two or one time points accordingly, therefore the study provides only a snapshot of gene regulation and chromatin binding by MPO. We have to admit that our study focused solely on ECs in a cell culture model, therefore, we cannot rule out the possibility that in a mouse model, other cell types may be affected, potentially leading to decreased vascularization as a result of combined MPO-triggered events.

## STAR★Methods

### Key resources table

**Table:** 

REAGENT or RESOURCE	SOURCE	IDENTIFIER
**Antibodies**		

Rabbit IgG (H+L) Alexa488	Thermo Fisher Scientific	A11034; RRID: AB_2576217
Mouse IgG (H+L) Alexa488	Thermo Fisher Scientific	A21042; RRID: AB_2535711
Rabbit IgG (H+L) Alexa647	Thermo Fisher Scientific	A21245; RRID: AB_2535813
Mouse IgG (H+L) Alexa647	Thermo Fisher Scientific	A32728; RRID: AB_2633277
Mouse IgG (H+L) Alexa555	Thermo Fisher Scientific	A32727; RRID: AB_2633276
Rabbit IgG (H+L) Alexa555	Thermo Fisher Scientific	A32732; RRID: AB_2633281
MPO	Calbiotech	475915; RRID: AB_212190
MPO	Thermo Fisher Scientific	RB373A; RRID: AB_59597
HISTONE H3	Santa Cruz	sc-8654; RRID: AB_2118303
GAPDH	Cell Signaling	2118I
ILF3	Abclonal	A2496; RRID: AB_2764387
CD31	Abcam	ab24590; RRID: AB_448167
CD34	Abcam	ab81289; RRID: AB_1640331
ICAM	Abcam	ab53013; RRID: AB_870702
CD45	Abcam	ab40763; RRID: AB_726545
Mouse IgG(H+L) HRP	Novex	A15981; RRID: AB_2534655
Rabbit IgG(H+L) HRP	Novex	A16023; RRID: AB_2534697
Goat IgG(H+L) HRP	Thermo Fisher Scientific	A15999; RRID: AB_2534673

**Chemicals, peptides, and recombinant proteins**		

Lipofectamine RNAiMAX	Thermo Fisher Scientific	13778075
Lipofectamine	Thermo Fisher Scientific	A12621
Direct-zol RNA miniprep kit	Zymo Research	R2051

**Critical commercial assays**		

ChIP-IT High Sensitivity kit	Active Motif	53040
Fluorescein (APF) assay	Thermo Fisher Scientific	A36003
Active Motif ATAC-Seq kit	Active Motif	53150

**Deposited data**		

Raw and analysed NGS data	This paper	[Gene Expression Omnibus] series GSE202015
Raw and analysed Mass spectrometry	This paper	PXD033079; Table S4

**Experimental models: Cell lines**		

HUVEC	Lonza	C2519A
HEK293T	Laboratory of Argyris Papantonis	N/A

**Experimental models: Organisms/strains**		

Mice: Mpo^-/-^ C57BL/6J	Jackson Laboratory, Bar Harbor, ME, USA	RRID:IMSR_JAX:004265

**Oligonucleotides**		

CD45 ACCACAAGTTTACTAACGCAAGT, TTTGAGGGGGATTCCAGGTAAT	PrimerBank	ID 18641362a1
VWF CCGATGCAGCCTTTTCGGA, TCCCCAAGATACACGGAGAGG	PrimerBank	ID 89191867c1
CXCL8 ACTGAGAGTGATTGAGAGTGGAC, AACCCTCTGCACCCAGTTTTC	PrimerBank	ID10834978a2
PPDPF TTCCACTTCCAGCAACAGCTC, AGAAGCTGGCCCACCAATGAC	Designed using NCBI Primer-blast – this paper	N/A
18srRNA CCCTATCAACTTTCGATGGTAGTCG, CCAATGGATCCTCGTT AAAGGATTT	Lal, Ashish et al.^37^	N/A
YWHAZ GCTTCACAAGCAGAGAGCAAAGT, TCTTGGTATGCTTGTTGTGACTG	Designed using NCBI Primer-blast – this paper	N/A
Mission siRNA Universal Negative Control #1	Sigma	SIC001
ILF3 siRNA1	Sigma	ILF3_1 PDSIRNA2D; SASI_Hs01_00242960
ILF3siRNA2	Sigma	SASI_Hs01_00242961

**Recombinant DNA**		

KA0637	Laboratory of Leo Kurian	RRID:Addgene_124166
Super piggyBac Transposase expression vector	SBI Biosciences	PB200PA-1
MPO-mVenus	This study	N/A
N-terminal-MPO-mVenus	This study	N/A
Middle-part-MPO-mVenus	This study	N/A
C-termina-MPO-mVenus	This study	N/A
C-terminal-part1-MPO-mVenus	This study	N/A
C-terminal-part2-MPO-mVenus	This study	N/A
C-terminal-part3-MPO-mVenus	This study	N/A
C-terminal-part4-MPO-mVenus	This study	N/A

**Software and algorithms**		

MACS2	Zhang, Y., Liu, T., Meyer, C.A. et al.^38^	https://github.com/macs3-project/MACS
HiChIP	Yan, H., Evans, J., Kalmbach, M. et al.^39^	https://bioinformaticstools.mayo.edu/
HOMER	Heinz S, Benner C, Spann N, Bertolino E et al.^40^	http://homer.ucsd.edu/homer/
Genrich		https://github.com/jsh58/Genrich
deepTools	Fidel Ramírez, Devon P Ryan, Björn Grüning, Vivek Bhardwaj, Fabian Kilpert, Andreas S Richter, Steffen Heyne, Friederike Dündar, Thomas Manke^41^	https://github.com/deeptools/deepTools
HINT-ATAC	Li, Z., Schulz, M.H., Look, T. et al.^42^	https://reg-gen.readthedocs.io/en/latest/hint/introduction.html
DESeq2	Love M.I., Huber W., Anders S.^43^	https://bioconductor.org/packages/release/bioc/html/DESeq2.html
InstantClue	Nolte, H., MacVicar, T.D., Tellkamp, F. et al.^44^	http://www.instantclue.uni-koeln.de/
Qupath	Bankhead, P. et al.^45^	https://qupath.github.io/
csaw R	Lun AT, Smyth GK.^46^	https://bioconductor.org/packages/release/bioc/html/csaw.html
GraphPad		https://www.graphpad.com/
Fiji	Schindelin, J., Arganda-Carreras, I., Frise, E. et al.^47^	https://fiji.sc/

**Other**		

POLR2A ChIP	ENCODE	https://www.encodeproject.org/experiments/ENCSR000EFB/
H3K27me3 ChIP	ENCODE	https://www.encodeproject.org/experiments/ENCSR000DVO/
H3K4me3 ChIP	ENCODE	https://www.encodeproject.org/experiments/ENCSR000DVN/
Control (input) for ChIP	ENCODE	https://www.encodeproject.org/experiments/ENCSR000EVV/
H3K4me3 ChIP	ENCODE	https://www.encodeproject.org/experiments/ENCSR000AKN/
H3K4me1 ChIP	ENCODE	https://www.ncbi.nlm.nih.gov/geo/query/acc.cgi?acc=GSM733690
H3K27ac ChIP	ENCODE	https://www.ncbi.nlm.nih.gov/geo/query/acc.cgi?acc=GSM733691
H3K27ac ChIP	ENCODE	https://www.ncbi.nlm.nih.gov/geo/query/acc.cgi?acc=GSM3557982
H3K4me3 ChIP	ENCODE	https://www.encodeproject.org/experiments/ENCSR000AKN/)

### Resource availability

#### Lead contact

Further information and requests for resources and reagents should be directed to and will be fulfilled by the lead contact, Yulia Kargapolova (y.kargapolova@imb-mainz.de).

#### Materials availability

All unique and stable reagents generated in this study are available from the lead contact with a completed materials transfer agreement.

#### Data and code availability

Data reported in this paper will be shared by the lead contact upon request. Proteomics data are available via ProteomeXchange with identifier PXD033079. Sequencing data are available via GEO with identifier GSE202015. This paper analyzes existing, publicly available data. The accession numbers for these datasets are listed in the key resources table. This paper does not report original code. Any additional information required to reanalyze the data reported in this paper is available from the lead contact upon request.

### Experimental model and study participant details

Animals used in the study were of C57BL/6J background (Jackson Laboratory, Bar Harbor, ME, USA). The study involved two groups consisting of 5 control and 5 MPO-deficient^48^ 8- to 12 week-old male, littermates were randomly assigned to experimental groups. The surgical procedure was conducted by a single surgeon, and all animals were housed in the same room within an animal facility to minimize variations in light exposure, ventilation, and other environmental factors. Outcome assessment imaging and quantification were carried out in a blinded manner to mitigate bias. All animal studies were approved by the local authorities (State Agency for Nature, Environment and Consumer Protection (LANUV), NRW, Germany) and conformed to the guidelines from Directive 2010/63/EU of the European Parliament on the protection of animals used for scientific purposes. All surgical interventions were performed under anaesthesia using isoflurane and perioperative analgesia with buprenorphine to minimize suffering.

The manuscript does not contain clinical studies or patient data.

### Method details

#### ChIP-seq and data analysis

HUVEC cells were pre-treated with 1 μg/ml of MPO for 2 or 8 hours; for control experiments fresh media was given to the cells. For each batch of ChIP experiments, ∼12 million cells were crosslinked in 2% PFA for 45 min at 4°C. Fixation was stopped by adding 1/20 media volume of Stop Solution. Cells were screped off the plate using a rubber policeman, centrifuged at 500 g at 4°C and washed twice in 10 ml of ice-cold PBS Wash buffer. From this point onward, cells were processed via the ChIP-IT High Sensitivity kit (Active motif) as per the manufacturer’s instructions, but using the NEXSON protocol for nuclei isolation.^49^^,^^50^ Namely, 3-5 pulses of low power settings were given sing Bioruptor Plus sonicator (Diageode; 30 s “on” and 30 s “off” at the low power setting). To ensure cell lysis was complete and the nuclei have been released, 10 μl of sonicated cell lysate was analysed under a phase contrast microscope using a hemocytometer. The nuclei were pelleted and the pellet resuspended in 500 μl of ChIP Buffer supplemented with 5 μl of PIC and 5 μl of 100 mM PMSF. Chromatin was sheared to 200–500-bp fragments on a Bioruptor Plus (Diagenode; 2× 20–26 cycles of 30 s “on” and 30 s “off” at the highest power setting). The 25 μl aliquot of shared chromatin was kept for analysis of shearing efficiency – same sample was used as “input” DNA for sequencing. The input DNA preparation has been carried out exactly as recommended by the kit manufacturer. The 1.5 % agarose gel has been used to visualize shared chromatin. The immunoprecipitations were carried out by adding 4 μg of the appropriate antibodies (MPO, Calbiochem) to ∼30 μg of chromatin (this amount was calculated by measuring the DNA yield resulting from the input DNA aliquot) and incubating on a rotator overnight at 4°C in the presence of protease inhibitors. Following addition of protein-A/G agarose beads and washing, DNA was purified using the ChIP DNA Clean & Concentrator kit (Zymo Research) and used in qPCR or sequencing on a HiSeq4000 platform (Illumina). Where ChIP-seq was performed, at least 20 million reads were obtained, also for the relevant “input” samples. Raw sequencing reads (typically 50 nt-long) were analyzed using the HiChIP pipeline,^39^ and peaks were called using MACS2.^38^ Thresholded MPO ChIP-seq peaks (q-value <0.05) per each cell type and genotype are listed in Table S1. For plotting ChIP-seq signal coverage over select regions, ngs.plot was used.^51^ The motif search around the MPO binding sites was performed as such: 1) windows of 200 bp of length were defined upstream and downstream of a peak center 2) motif analysis was performed for upstream and downstream selected regions separately, using HOMER software (Heinz et al., 2010) by running findMotifsGenome.pl command; results of *de-novo* motif discovery were used.

#### HUVEC culture and MPO treatment, MPO inactivation

Human Umbilical Vein Endothelial Cells, pooled from several donors, were purchased from Lonza and cultured in FBS reduced Endopan 3 media. HUVEC cells of passages 5-10 were used for experiments, when cells were actively proliferating. Culturing conditions were 37°C and 5% CO2 with saturating humidity – control experiments were performed in parallel to MPO treatments with the cells cultured to the same cells density. MPO treatment was performed with protein purified from human blood (Planta) and at 1 μg/ml final concentration. MPO was inactivated as previously described by Paumann-Page and colleagues.^52^ In brief, 100 μg of MPO was incubated in 0.5 M H_2_O_2_ 30 min at 37°C, remaining H_2_O_2_ was removed by dialysis. Loss of enzymatic activity was confirmed by 3’-(p-aminophenyl) Fluorescein (APF) assay (ThermoFisher).

#### Identification of nuclear localizing domain of MPO

Doxicycline-inducible overexpression HEK293T cells were generated using PiggyBac transposition. The N-terminal (1-252 a.a.) middle (253 - 509 a.a.) and C-terminal (510 - 747 a.a.) parts of MPO were cloned into KA0717 vector via MluI/SpeI restriction sites. C-terminal part of MPO was further fragmented into 68 a.a, 67 a.a, 44 a.a, 58 a.a. long peptides. Following validation by Sanger sequencing, HEK293T cells were co-transfected together with transactivator and transposase-encoding vectors (KA0637 and SBI Biosciences #PB200PA-1, respectively) at a DNA mass ratio of 10:1:3 using Lipofectamine LTX (ThermoFisher) as per manufacturer’s instructions. Stable, transgene-positive HEK239T cells were selected using 250 μg/ml G418 (Sigma Aldrich). For imaging, transfected cells were grown on coverslips and overexpression was induced by 16h treatment with Doxycycline. After induction, the cells were fixed in 4% PFA/PBS for 15 min at room temperature, washed with PBS and permeabilized with 0.5% Triton-X/PBS for 5 min at room temperature. The coverslips were mounted in slides with ProLong™ Gold Antifade Mountant with DAPI (ThermoFisher). Images were acquired on a Leica DMI8 Inverted Fluorescence Phase Contrast Microscope and on an Olympus Fluoview 1000 Spectral-based Laser Scanning Confocal Microscope.

#### Assay for transposase-accessible chromatin using sequencing (ATAC-seq) and data analysis

ATAC-Sequencing was performed with help of Active Motif ATAC-Seq kit as per manufacturer instructions. HUVEC cells were pre-treated with 1 μg/ml of MPO for 2 or 8 hours; for control experiments media was changed accordingly. Cells were lifted up with Trypsin and counted, 50,000 to 100,000 cells used per experiment. First, cells were pelleted by centrifugation at 500x g for 5 minutes, at 4°C, washed in ice-cold PBS, and pelleted again. Next, cell pellet was resuspended in ice-cold ATAC Lysis Buffer and transferred into a PCR tube. After another round of centrifugation, cell pellet was resuspended in the Tagmentation Master Mix, assembled exactly as recommended by the manufacturer, namely in 50 μl of total reaction volume containing 25 μl 2x Tagmentation Buffer, 2 μl 10x PBS, 0.5 μl 1.0% Digitonin, 0.5 μl 10% Tween 20, 12 μl H_2_O and 10 μl of Assembled Transposomes. The tagmentation reaction was performed at 37°C for 1 hour. Further, tagmented DNA was purified. Library preparation was performed as suggested by kit manufacturer. Ready libraries were purified with SPRI clean-up beads. Sequencing was performed in paired-end way and 100 bp per read. Reads were mapped to human genome version hg38. Peak calling was performed with help of MACS2 and Genrich (https://github.com/jsh58/Genrich), differential chromatin accessibility was assessed by csaw R package as described by Reske et al.^53^ To estimate nucleosome positioning around the MPO peaks, reads were sub grouped by length using deepTools package^41^ and bamcoverage option with length windows of 10 to 50 bp for short free DNA, 51 to 100 for long free DNA and 180 to 247 for mononucleosomes, exact scaling and RPKM normalization options on. Identification of transcription-binding sites from ATAC-Seq data was performed using HINT-ATAC workflow.^42^

#### Generation and analyses of total RNA-seq

Cells at 2 different time points after treatment with MPO were harvested in Trizol (Life Technologies) and total RNA was isolated and DNase-treated using the Direct-zol RNA miniprep kit (Zymo Research) as per the manufacturer’s instructions. Barcoded cDNA libraries were generated using the TruSeq RNA library kit (Illumina) via selection on poly(dT) beads. The resulting libraries were paired-end sequenced to >50 million read pairs on a HiSeq4000 platform (Illumina). Reads were trimmed and mapped to human genome hg19 using STAR; Reads uniquely mapping to exons were quantified using HTSeq-count and differential gene expression was assessed using DESeq2.^43^ Differentially-regulated genes per each cell line and treatment are listed in Table S3.

#### MPO immunoprecipitation and proteomics

Immunoprecipitation was performed from isolated nuclei (after 8 hours MPO treatment at a concentration 1 μg/ml), non-treated cells were used as negative control. For immunoprecipitation antibody against MPO (Calbiochem, 475915) was used for both treated and untreated cells. Cell nuclei were isolated by incubating cells for 15 min on ice in NIB buffer (15 mM Tris-HCL pH 7.5, 60 mM KCl, 15 mM NaCl, 5 mM MgCl_2_, 1 mM CaCl_2_, 250 mM sucrose) containing 0.3% NP-40 as previously described in.^50^ Nuclei were pelleted for 5 min 800 × g at 4°C, washed twice in the same buffer, lysed for 10 min on ice in IP buffer (150 mM LiCl, 50 mM Tris-HCl pH 7.5, 1 mM EDTA, 0.5% Empigen) freshly supplemented with 2 mM sodium vanadate, 1× protease inhibitor cocktail (Roche), PMSF (10 μl), 0,5 mM DTT, and 50 units Benzonase per ml of IP buffer, before preclearing cell debris by centrifugation at >15,000 × g at 4°C. Finally, 1 mg of the lysate was incubated with anti-MPO antisera overnight at 4°C. Magnetic beads (Active Motif; ChIP-IT Protein G Magnetic Beads) were then washed once with 1× PBS-Tween and combined with the antibody-lysate mixture. Following a 2h incubation at 4°C, beads were separated on a magnetic rack and washed 5×, 5 min each in wash buffer (150 mM KCl, 5 mM MgCl_2_, 50 mM Tris-HCl pH 7.5, 0.5% NP-40) and another two times in wash buffer without NP-40. Captured proteins were pre-digested and eluted from the beads using digestion buffer (2 M Urea, 50 mM Tris-HCl pH 7.5, 1 mM DTT) supplemented with trypsin and eluted from the beads with elution buffer (2 M Urea, 50 mM Tris-HCl pH 7.5, 5 mM chloroacetamide) supplemented with trypsin and LysC, before subjected to mass-spectrometry on a Q-Exactive Plus Orbitrap platform coupled to an EASY nLC (Thermo Scientific). Peptides were loaded in solvent A (0.1% formic acid in water) onto an in-house packed analytical column (50 cm length, 75 μm I.D., filled with 2.7 μm Poroshell EC120 C1; Agilent); were chromatographically separated at a constant flow rate of 250 nL/min using the following gradient: 3–8% solvent B (0.1% formic acid in 80% acetonitrile) for 1 min, 8–30% solvent B for 39 min, 30–50% solvent B for 8 min, 50–95% solvent B for 0.3 min, followed by washing and column equilibration. The mass spectrometer was operated in data-dependent acquisition mode. An MS1 survey scan was acquired from 300–1750 m/z at a resolution of 70,000. The top 10 most abundant peptides were isolated within a 1.8 Th window and subjected to HCD fragmentation at a normalized collision energy of 27%. The AGC target was set to 5e5 charges, allowing a maximum injection time of 110 ms. Product ions were detected at a resolution of 35,000; Precursors were dynamically excluded for 10 s. All raw data were processed with Maxquant (v1.5.3.8) using default parameters. Briefly, MS2 spectra were searched against the Uniprot HUMAN fasta (16.06.2017) database, including a list of common contaminants. False discovery rates on were estimated by a target-decoy approach to 1% (Protein FDR) and 1% (PSM FDR), respectively. The minimal peptide length was set to 7 amino acids and carbamidomethylation at cysteine residues was considered as a fixed modification. Oxidation (M) and Acetyl (Protein N-term) were included as variable modifications. The match-between runs option was enabled and LFQ quantification was enabled under default settings. The full list of peptide hits and their analysis is provided in Table S4.

#### Immunostaining and imaging

Cells were grown on coverslips, fixed in 4% PFA/PBS for 10 min at room temperature, washed in 1× PBS, permeabilized in 0.5% Triton-X/PBS for 5 min at room temperature, blocked in 1% BSA/PBS for 1 h before incubating with the primary antibody of choice for 2 h to overnight. Cells were next washed twice in 1x PBS for 5 min, before incubating with the appropriate secondary antisera for 1 h at room temperature. Nuclei were counterstained with DAPI (Sigma-Aldrich) for 5 min, washed, and coverslips mounted onto slides in Prolong Gold Antifade (Invitrogen). For image acquisition, a fluorescent microscope Olympus IX83, 60× (Oil) objective was used, making sure exposure times were maintained constant across samples in each imaging session for the same immunostaining. Finally, images were analyzed using the Fiji suite^47^ as follows: first, background signal levels were subtracted using the embedded function (rolling ball function of 50-px radius with a sliding paraboloid and disabled smoothing), and the DAPI channel was used to determine the area of interest where signal would be quantified from. Measured mean signal intensities were used to generate plots in R or via InstantClue.^44^ For bean plots, dots represent the mean of the dataset; for box plots, whiskers’ ends represent the top and bottom quantiles, respectively.

#### Nuclei and chromatin fractionation and western blotting

For nuclei fractionation HUVECs from 15 cm plates were washed in 10 ml/ plate ice-cold PBS and collected by centrifugation (3000g, 3 min, 4°C). Collected cells were washed with PBS, pelleted and lysed in 1 ml ChIP prep buffer ChIP-IT High Sensitivity kit, Active Motif), supplemented with protease inhibitor cocktail and PMSF. Lysis was performed 10 minutes on ice, following mild sonication as described in NEXSON protocol for nuclei isolation.^49^ Namely, 3-5 pulses of low power were given using Bioruptor Plus sonicator (Diageode; 30 s “on” and 30 s “off” at the low power setting). To ensure cell lysis was complete and the nuclei have been released, 10 μl of sonicated cell lysate was analysed under a phase contrast microscope using a hemocytometer. Nuclei were collected via centrifugation for 3 min at 1250 g at 4°C and washed x1 with 1 ml of ChIP prep buffer.

For chromatin fractionation HUVECs were grown to 80-90% confluency, washed with PBS and Trypsinized. Trypsin was inactivated by adding 10% FBS containing media. Cells from x 10 cm dishes were pelleted and lysed in 300 μl of chromatin extraction buffer (20mM Tris, pH 7.5, 100mM NaCl, 5 mM MgCl_2_, 10% glycerol, 0.2% NP-40, 1mM NaF, 0.5 mM CTT, 1x complete protease inhibitor and phosphatase inhibitor (Roche). Lysate was sonicated using Active Motif dounce homogenizer, with 3 pulses at 25% intensity 30 sec on and 30 sec off. Cell debris was pelleted for 5 minutes at 2500 rpm at 4°C, supernatant was collected. Supernatant from previous step was centrifuged 10 minutes at 13,000g at 4°C, pellet was washed 3 times using chromatin extraction buffer, this fraction constitutes the chromatin fraction. The supernatant was collected and constitutes soluble fraction. Chromatin fraction was resuspended in 50 μl TBS/Tween with addition 5 μl of benzonase (500 kU/μl, Merk Millipore) and incubated at 37°C 30 minutes, following western blot analysis.

Western blotting was performed as previously described.^54^ Namely, ∼2 × 10^6^ cells were enzymatically detached or gently scraped off cell culture dishes, and pelleted for 5 min at 600 × g. The supernatant was discarded, and the pellet resuspended in 100 μl of ice-cold RIPA lysis buffer (150 mM NaCl, 1% NP-40, 0.5% DOC, 0.1% SDS, 50mM Tris (pH 7.4) containing 1× protease inhibitor cocktail (Roche), incubated for 30 min on ice, and centrifuged for 15 min at >15,000 × g to pellet cell debris and collect supernatant. Total protein concentrations were determined using the Pierce BCA Protein Assay Kit (ThermoFisher Scientific). Lysates were stored at −80°C. Proteins were resolved by SDS-PAGE, transferred onto membranes using the TransBlot Turbo setup (Bio-Rad), and detected using the antibodies listed in the KRT.

#### MNase treatment and gel electrophoresis, *in vitro* migration assays

HUVECs were grown in 6-well plates to ∼80% confluency and rinsed with PBS prior to addition of 1 ml of freshly prepared permeabilization buffer (15 mM Tris/HCl pH 7.6; 60 mM KCl; 15 mM NaCl; 4 mM CaCl_2_; 0.5 mM EGTA; 300 mM sucrose; 0.2% NP-40; 0.5 mM β-mercaptoethanol supplemented with MNase (Sigma) at 1 μ/ml final concentration. MNase was added for 2, 4 and 6 min at 37°C, and stopped by addition of an equal volume of stop buffer (50 mM Tri/HCl pH 8.0; 20 mM EDTA; 1% SDS). Finally, 250 μg RNase A were added for 2 h at 37°C, followed by addition of 250 μg proteinase K and incubation at 37°C overnight. Next day, DNA was isolated via standard phenol-chloroform extraction, digestion efficiency was determined after electrophoresis in 1% agarose gels. Images were quantified using Fiji suite.

For *in vitro* migration (scratch) assays, HUVECs were grown in 6-well plates and one scratch per well was manually inflicted using a sterile cell scraper. Cell migration into the scratch was monitored for up to 20 h by bright field microscopy.

#### Electromobility shift assay

Nucleosomes were reconstituted as previously described by Luger et al.^55^ and Dyer et al.^56^ Binding of reconstituted nucleosomes with MPO was performed 30-45 minutes in total volume of 10 μl of binding buffer (25mM HEPES, 150 mM KCl) at room temperature. Titers of MPO varying from 50 nM to 1000 nM were used to bind with 100 nM nucleosomes with short and long linker DNA. Sucrose solution at a final concentration of 5% was added as a loading buffer prior to sample loading. 5% TBE native polyacrylamide gel was used for analysis. Gel was pre-run without samples at 150 V for 60 minutes in the cold room on ice, the run with samples was performed with same voltage settings, for 50 minutes. Gel was stained in 50 ml TBE with 1:10,000 diluted SYBR gold for exactly 10 minutes, washed with water and visualized using GelDoc imaging System (Bio-Rad).

#### siRNA experiments ILF3 KD, 3’end sequencing, transcription inhibition by DRB

Universal negative control siRNA (Mission siRNA Universal Negative Control #1, SIC001, Sigma) and ILF3 siRNA1 and ILF3siRNA2 (ILF3_1 PDSIRNA2D, SASI_Hs01_00242960, SASI_Hs01_00242961, Sigma) were used in ILF3 knockdown experiments. RNAiMAX (ThermoFisher) and Opti-MEM (ThermoFisher) were used for transfection. To ensure an ideal knockdown rate, HUVECs were cultured in complete Endopan3 medium on 6-well plates (overnight), and shifted to no-antibiotic Endopan3 medium for siRNA transfection. Transfection was done according to the protocol provided by ThermoFisher. Knockdown efficiency was assessed by qPCR 24 hours and by western blot 72 hours after transfection. 3’-end RNA-seq was used as a lower-cost alternative to mRNA-seq. Libraries were prepared from total RNA using the QuantSeq 3’ mRNA-Seq Library Prep Kit (Lexogen), and paired-end sequenced on a HiSeq4000 platform (Illumina) generating ∼15 × 10^6^ 100 nt-long reads per sample. Reads were quality assessed and mapped to hg19 using STAR.^57^ Reads uniquely mapping to exons were quantified using HTSeq-count and differential gene expression was assessed using DESeq2.^43^ Differentially-regulated genes per each cell line and treatment are listed in Table S5. DRB treatment was performed in triplicates, for 30 min or 1 hour on ECs, cultured on 6-well plates. 3.1 mM DRB (TCI chemicals) stock solution in DMSO was applied to ECs at final concentration of 0.1 mM. Prior to DRB treatment cells were treated with siRNA against ILF3 or control siRNA for 71 hour, MPO treatment was performed for 2 hours at a standard concentration of 1 μg/ml. Cells were collected at 72 hours post-transfection, RNA was isolated, subjected to cDNA synthesis and qPCR.

#### Left anterior descending artery ligation and DAB staining

LAD ligation was performed as previously described.^58^ Mice were anesthetized with isoflurane, received low dose buprenorphine subcutaneously (Essex-Pharma, Munich, Germany; 0.05 mg/kg bodyweight) for analgesia and were placed on a heating pad to regulate body temperature. Following endotracheal intubation, animals were ventilated with 150 strokes/min and stroke volume of 7 μl/g bodyweight (Harvard Apparatus, Holliston, MA, USA). Surgical procedures were carried out using a dissecting microscope (Leica MZ6, Leica Microsystems, Wetzlar, Germany). After lateral thoracotomy of the fourth intercostal space, a suture (8/0 polypropylene suture, Polypro, CP Medical, Norcross, GA, USA) was placed around the LAD and the artery was ligated with a bow tie. Ischemia was visually confirmed by blanching of the left ventricular (LV) apex. Mice were sacrificed 8 weeks after infarction, hearts were processed for DAB staining using DAB staining kit (Abcam, ab64238) and following the manufacturer recommendations, at least 50 images per mouse were analysed using Qupath software.^45^

### Quantification and statistical analysis

P-values associated with Student’s/Welch t-tests and Analysis of variance (ANOVA) tests were calculated using GraphPad [http://graphpad.com. Unless otherwise stated, only P-values <0.05 were deemed as significant. Also, note that for immunofluorescence analyses, representative images from one of at least two independent and converging experiments are displayed and quantified.

## Supplemental information

Document S1. Figures S1–S5

Table S1. MPO and iMPO ChIP-Seq, overlap with regulated gene set

Table S2. ATAC-seq regions of increased chromatin / decreased chromatin accessibility

Table S3. RNA-Seq 2h MPO, 8h MPO, Q91T, TNF-alpha

Table S4. MPO Co-IP MS data, protein phosphorylation analysis

Table S5. 3′ end sequencing data
