# Supplementary material for: Remodeling of the endothelial cell transcriptional program via paracrine and DNA-binding activities of MPO
Source: iScience. 2024 Jan 12;27(2):108898. doi: 10.1016/j.isci.2024.108898 (PMC10844825; doi:10.1016/j.isci.2024.108898)
Supplement: Document S1. Figures S1–S5 [file mmc1.pdf]

iScience, Volume *27*

Supplemental information

Remodeling of the endothelial cell transcriptional program via paracrine and DNA-binding activities of MPO

Ruiyuan Zheng, Kyle Moynahan, Theodoros Georgomanolis, Egor Pavlenko, Simon Geissen, Athanasia Mizi, Simon Grimm, Harshal Nemade, Rizwan Rehimi, Jil Bastigkeit, Jan-Wilm Lackmann, Matti Adam, Alvaro Rada-Iglesias, Peter Nuernberg, Anna Klinke, Simon Poepsel, Stephan Baldus, Argyris Papantonis, and Yulia Kargapolova

Supplementary Figures

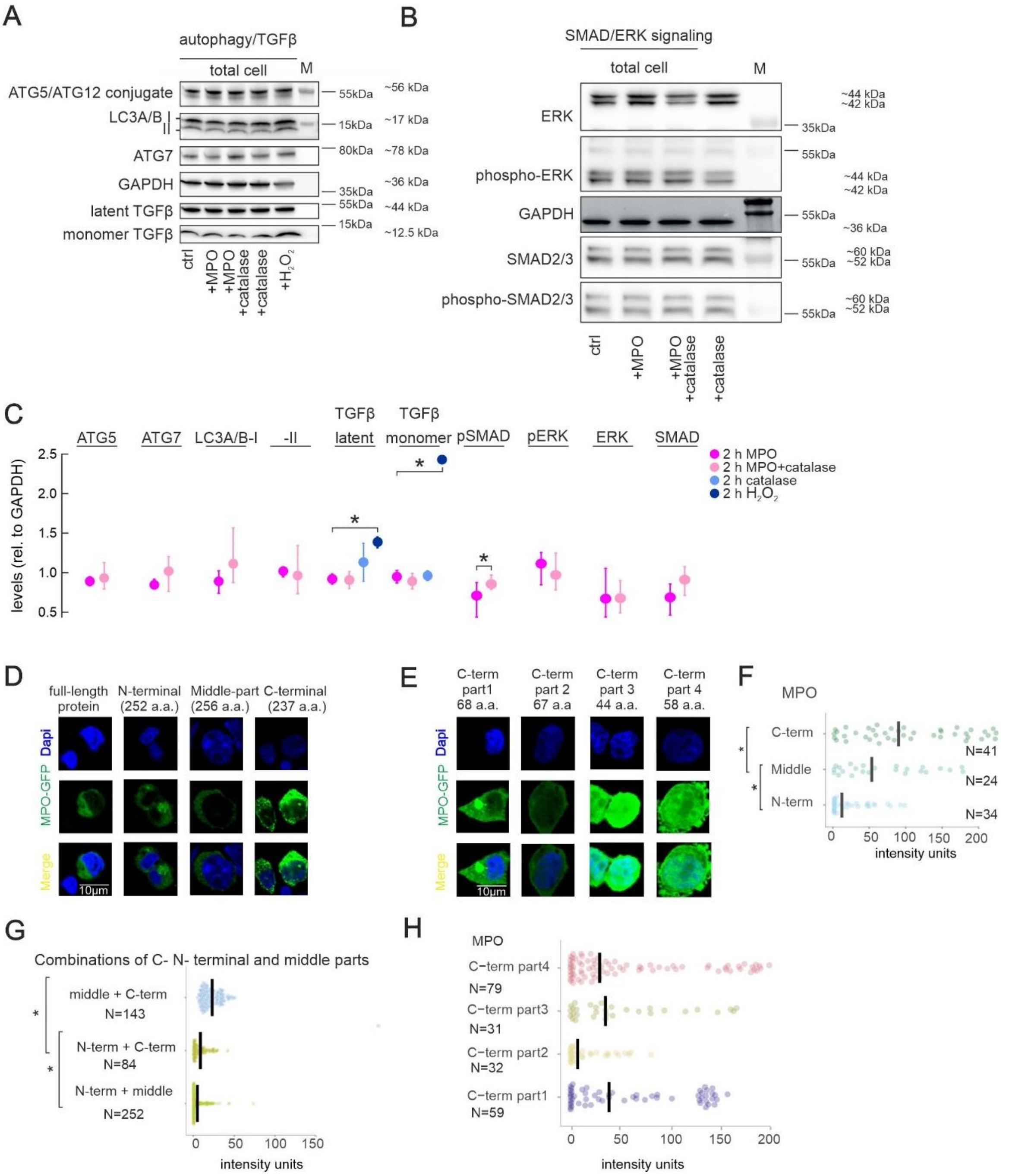

Figure S1. MPO translocates into the cytoplasm and nuclei of endothelial cells, Related to Figure 1.

(A) Western blot represents levels of ATG5-ATG12, LC3A/B, ATG7, GAPDH, latent TGFß and monomer TGFß upon MPO, MPO and catalase, catalase and H_2O_2 (used as positive control) treatments in ECs, representative images of three biological replicates. M – protein ladder.

(B) Western blot represents levels of SMAD2/3, phospho-SMAD2/3, ERK, phospho-ERK, GAPDH upon 2 hour MPO treatment or combination of MPO and catalase of ECs, representative image of three replicates.

(C) Relative levels of protein depicted on Figure S1A and S1B, normalized to GAPDH, data are presented as mean ±SEM. Statistical significance was determined by Welch's t-test.

(D) Fluorescence microscopy images of stable HEK293T cell lines inducibly overexpressing full-length and fragmented MPO-mVenus fusion proteins.

(E) Fluorescence microscopy images of stable HEK293T cell lines inducibly overexpressing C-terminal parts of MPO fused with mVenus.

(F) Quantification of mean nuclear fluorescence of mVenus-fused to C-terminal, middle and N-terminal MPO parts. Statistical significance was determined by two-sample t-test.

(G) Quantification of nuclear mVenus signal in stable HEK293T cell lines inducibly overexpressing combinations of middle and C-terminal, N-terminal and C-terminal, N-terminal and middle parts of MPO. Statistical significance was determined by two-sample t-test.

(H) Quantification of nuclear mVenus signal in stable HEK293T cell lines inducibly overexpressing fragments of C-terminal part of MPO (shown on (E)).

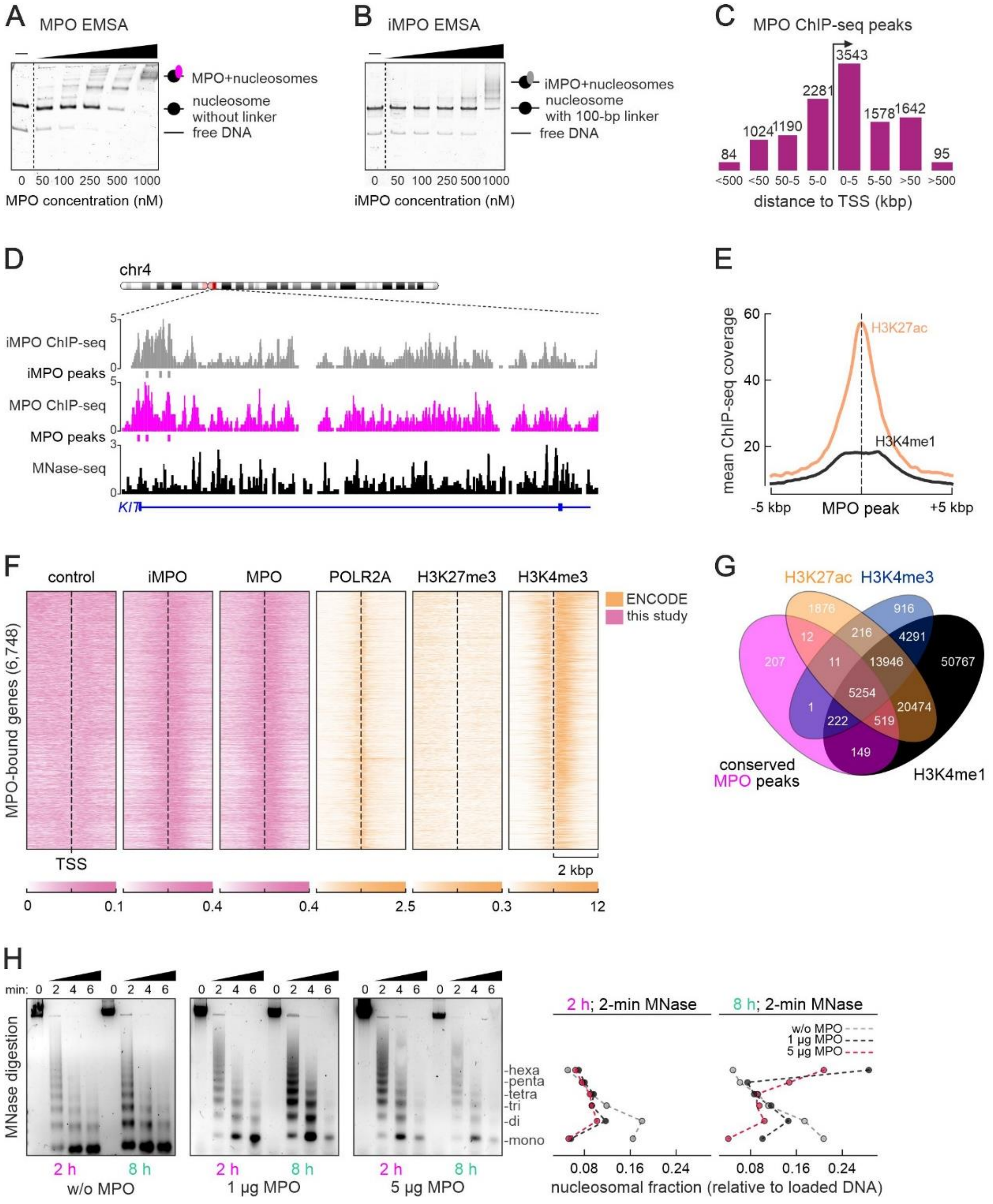

Figure S2. Binding of Myeloperoxidase to chromatin, sites distribution and chromatin condensation, Related to Figure 2.

(A) DNA EMSAs performed with increasing titers of MPO with 'no linker' DNA nucleosomes.

(B) DNA EMSAs performed with increasing titers of inactivated iMPO and long linker DNA nucleosomes (100bp).

(C) Barplots showing distribution of MPO binding sites around transcription start sites (TSS) of bound genes.

(D) Representative genome browser view of MPO and iMPO ChIP-Seq in MPO/iMPO-treated HUVEC cells (upper panels, current work) and MNase-Seq experiment by Diermeier and colleagues [24].

(E) H3K27ac and H3K4me1 ChIP-Seq (ENCODE) coverage centered at around MPO peaks shows high levels of H3K27ac signal.

(F) Heatmaps showing input, iMPO, MPO (current study), POLR2A, H3K27me3, H3K4me3 (ENCODE) ChIP-Seq signal in the 4 kbp windows around MPO-bound TSS.

(G) Venn diagram depicts an overlap between conserved MPO peaks (reproducible between two biological replicates), H3K27ac, H3K4me3 and H3K4me1 marks.

(H) Agarose gel images resolve MNase-treated DNA fragments, corresponding to mono-, di-, tri-, tetra, penta- and hexa-nucleosomes after 2 and 8 h MPO treatment or mock treatment. The abundance of each sort of fragments is quantified and normalized to the total DNA loaded onto the gel, and plotted as a line plot.

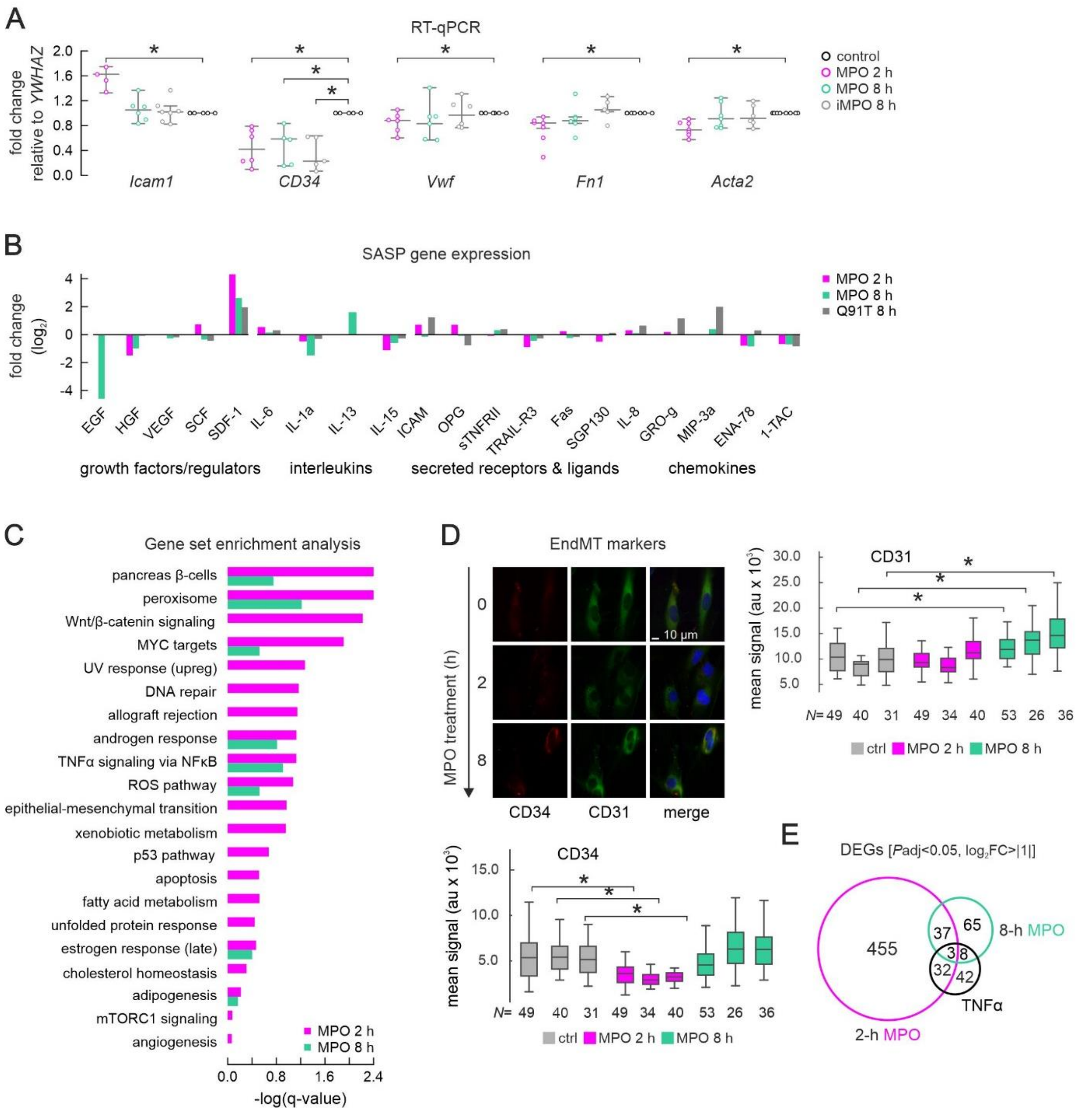

Figure S3. Signalling pathways regulated after 2 and 8 h of MPO treatment, Related to Figure 3.

(A) Relative expression of *Icam1*, *CD34 Vwf*, *Fn1* and *Acta2*, upon MPO (2h and 8h) and iMPO (8h) treatment, measured by qPCR and normalized to housekeeping transcript *Ywhaz* and relative to non-treated cells. Data are presented as mean ±SEM, asterisk labels Welch-test signif. pval<0.05.

(B) Gene expression changes (RNA-Seq) of a subset of genes, grouped as growth factors, interleukins, secreted ligands, chemokines, represented as bars, showing log2 fold changes between wild type MPO (2 and 8 h) or Q91T mutant MPO (8h) treated samples and controls.

(C) Gene set enrichment analysis (GSEA) of ranked gene expression data, generated by RNA-Seq of HUVECS under 2 and 8 hour MPO treatment and control (fresh media) treatment. Bar plots represent –log10 FDR q-values of GSEA results for 2 (pink) and 8 (green) hour MPO-treated samples.

(D) Representative immunofluorescence images of markers of Endothelial-to-Mesenchymal transition CD31and CD34 in ECs after 0, 2 and 8 hours of treatment, scale bar=10µm. Treatments performed in triplicates and violin plots depict quantifications of 10-15 random images selected

for each biological replica. Data are presented as mean ±SEM. Statistical significance was determined by two-sample t-test.

(E) Venn diagrams showing an overlap between differentially expressed genes after 2 and 8 hours MPO and TNFα treatment [24].

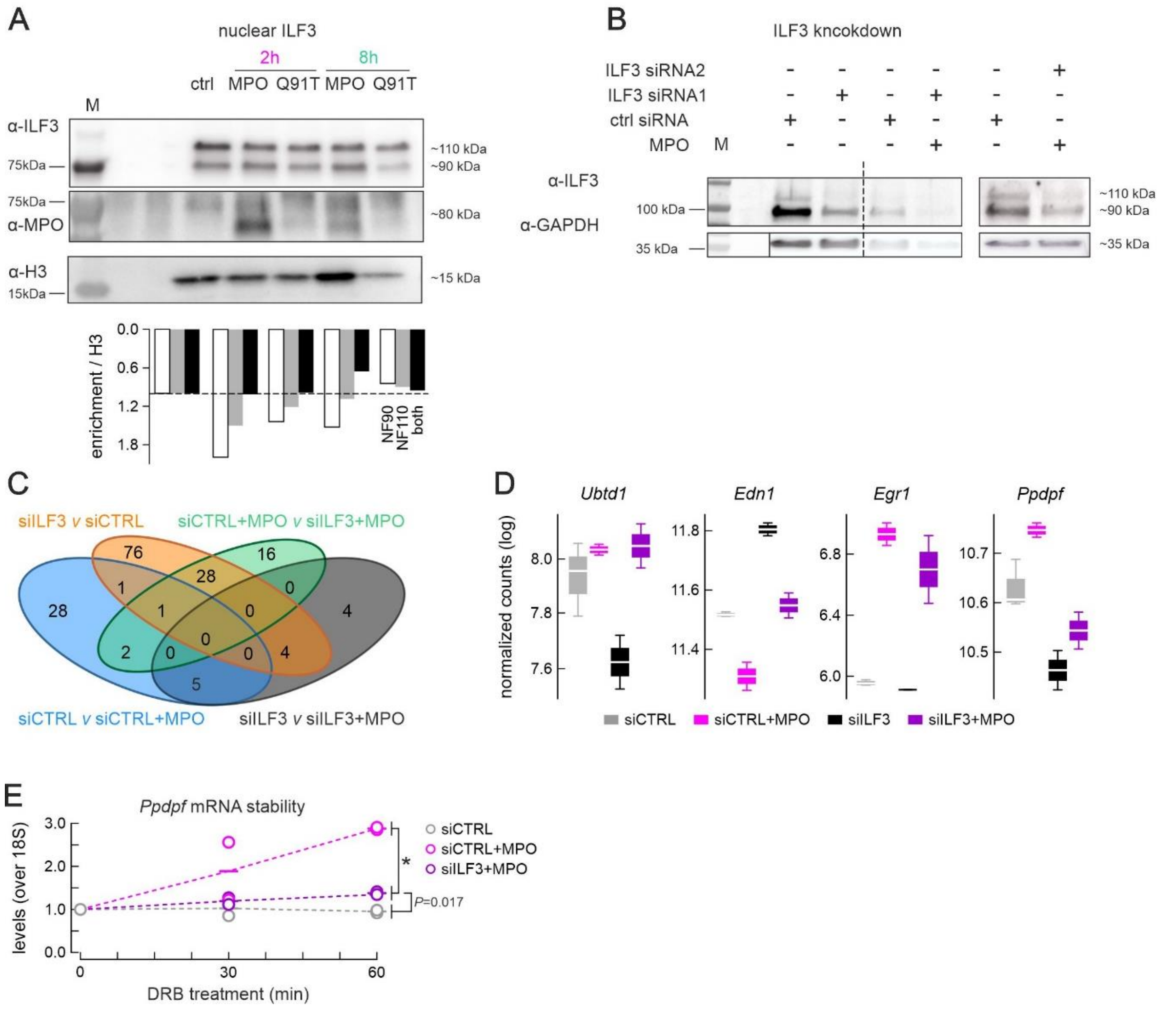

Figure S4. MPO treatment triggers changes in ILF3/NF90 levels in cytoplasm and nucleus and empowers regulation of ILF3-bound mRNAs, Related to Figure 4.

(A) Western blot image performed on nuclear fraction of ECs upon treatment with MPO or Q91T mutant MPO variant, and showing ILF3/NF90 increment in the nucleus post- treatment. Quantification of Western blot, normalized to histone H3 levels (lower panel). M – protein ladder.

(B) Western blot image, controlling for high efficacy of ILF3 depletion, performed with 2 different siRNAs against ILF3 and accompanied by MPO treatment prior to 3'-end Sequencing experiment. M – protein ladder.

(C) Venn diagram shows an overlap between 4 gene sets (generated by 3'-end RNA-sequencing) including differentially expressed upon MPO treatment and with or without ILF3 depletion.

(D) Box plots show mean regularized log transformed counts (generated by 3'-end RNA-sequencing) for a subset genes, whose expression is regulated by ILF3 depletion and restored by co-treatment with MPO (*UBTD1*, *EDN1*), and a subset of genes regulated by MPO independently of ILF3 (*EGR1*) or restored by combination of MPO treatment with ILF3 depletion (*PPDPF*). Data are presented as mean ±SEM.

(E) Stability assay performed with help of RT-qPCR and after inhibition of transcription with 5,6-Dichlorobenzimidazole 1-beta-D-ribofuranoside (DRB) for 1 hour time, detecting levels of *Ppdpf* transcript, and normalized by 18S. Statistical significance was determined by Welch's t-test.

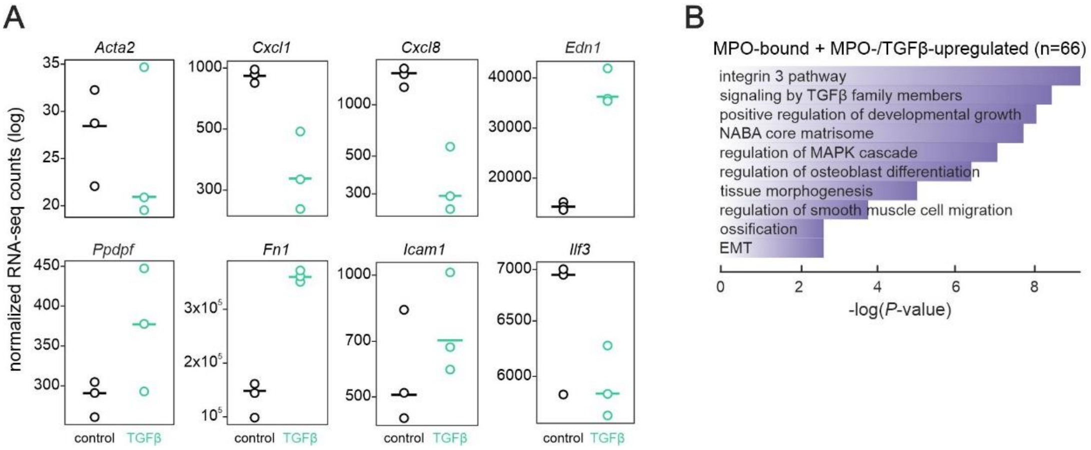

Figure S5 Overlapping gene regulation by MPO and TGFβ are bound by MPO and involved in EMT, Related to Figure 5.

(A) Dotplot, showing differential expression of selected transcripts after TGFβ treatment in HUVECs.

(B) Metascape analysis of genes bound by MPO (+/- 1000 bp from the peak center) and regulated by either MPO or TGFβ or both.
